# Wearable Sensor-Based Daily Life Walking Assessment of Gait for Distinguishing Individuals With Amnestic Mild Cognitive Impairment

**DOI:** 10.3389/fnagi.2019.00285

**Published:** 2019-10-22

**Authors:** Haiqun Xie, Yukai Wang, Shuai Tao, Shuyun Huang, Chengguo Zhang, Zeping Lv

**Affiliations:** ^1^Department of Neurology, First People’s Hospital of Foshan, Foshan, China; ^2^Dalian Key Laboratory of Smart Medical and Health, Dalian University, Dalian, China; ^3^National Research Center for Rehabilitation Technical Aids, Rehabilitation Hospital, Beijing Key Laboratory of Rehabilitation Technical Aids for Old-Age Disability, Key Laboratory of Intelligent Control and Rehabilitation Technology of the Ministry of Civil Affairs, Beijing, China

**Keywords:** amnestic mild cognitive impairment, gait, wearable sensor-based, cognition, gait parameters

## Abstract

**Objectives**: To characterize gait disorders in patients with amnestic mild cognitive impairment (aMCIs) and determine the association between the performance of the gait function and cognition.

**Methodology**: In this study, we enrolled 38 patients with aMCI and 30 cognitively normal individuals normal controls (NC). Neuropsychological assessments included tests of memory, executive function, language, and attention. Using an inertial-sensor-based wearable instrument, we collected the gait data dynamically for at least 1 h/day for 2 weeks. The gait parameters included walking velocity, stride length, stride time, cadence, and stride time variability.

**Results**: The aMCI patients had reduced walking velocity and stride length and increased stride time variability compared with the NCs. The total number of steps, stride time, and cadence did not differ between the two groups. For all the subjects, walking velocity and stride length was positively associated with memory and executive function. Stride time variability was negatively correlated with the cognitive domains including memory, executive function and attention.

**Conclusion**: This study suggested that cognitive impairment-related gait disorders occur (reduced gait speed, gait length, and gait stability) in daily life walking among the aMCI patients. A sensor-based wearable device for gait measurement may be an alternative and convenient tool for screening cognitive impairment.

## Introduction

Amnestic mild cognitive impairment (aMCI) is a subtype of MCI (Petersen, 2004). It can present as a prodromal stage of Alzheimer’s disease (AD; Albert et al., 2011; Morris, 2012). Early diagnosis and intervention for aMCI are urgently needed. The standard diagnostic procedure for aMCI primarily relies on a comprehensive neuropsychological assessment that explores various cognitive domains, combined with blood tests and brain imaging. An etiological diagnosis of MCI due to AD is performed using well-established neural biomarkers derived from CSF or/and PIB-PET brain amyloid imaging (Albert et al., 2011; Knopman et al., 2018). However, these methods applied for diagnostic process are time-consuming, expensive, and restricted in availability Recently, alternative readily available biomarkers have been explored for briefly screening cognitive impairment and monitoring the disease progress of AD (Laske et al., 2015). Among these, the gait parameter has been recognized as a potentially promising biomarker.

Previous studies have shown that motor dysfunction occurs in individuals with declined cognitive function (Mielke et al., 2013; Beauchet et al., 2016; Montero-Odasso et al., 2017). In particular, growing evidence indicates that quantitative assessment of spatio-temporal gait parameters including velocity, stride length, stride time, and variability of stride time (STV) was worse among individuals with MCI than among healthy controls under single-task conditions (Beauchet et al., 2013; Allali et al., 2016; Bahureksa et al., 2017). Deficits in cognitive function and mobility co-exist in older adults with MCI in either the memory or the non-memory domains(Beauchet et al., 2014; Doi et al., 2014; Ansai et al., 2018). Impairments in cognitive domains such as attention, executive function, and memory are associated with reduced gait velocity and gait instability (Beauchet et al., 2014; Doi et al., 2014; Verlinden et al., 2014; Ansai et al., 2018). Longitudinal studies have suggested that gait disorder preceded cognitive decline and could be a marker to identify individuals with MCI at the risk of developing AD early (Gillain et al., 2016; Kikkert et al., 2016; Montero-Odasso et al., 2017). However, most of the previous studies recruited subjects with MCI, which was heterogeneous including the subtypes of either amnesic or non-amnestic MCI. Evidence of the association of amnesic MCI, which most often progresses to AD dementia, with gait dysfunction is limited. Further studies are required to evaluate the gait parameter as an indicative and predictive biomarker of incident AD among individuals with aMCI.

Traditionally, a quantitative measurement for gait has been based mainly on video images of the subjects walking and complex noise preprocessing, motion detection, and motion segmentation. It is generally expensive and only allows for measurements in a strict experimental setting. In recent years, the instruments and technology for the quantification of gait performance have increasingly improved. A sensor-based wearable device is one of the attractive methods developed and allows for a quantitative and continuous assessment of the gait performance (Tao et al., 2018; Micó-Amigo et al., 2019). It is convenient for daily life, either indoor or outdoor use, and long-term monitoring. Compared with the conventional one-time in-lab measurement, continuously collecting the gait parameter data in daily life may more objectively reflect a subject’s mobility function and gait pattern, thus providing a higher technical guarantee and potential value to explore the linkage between cognitive impairment and gait disturbance. By means of this technology, several studies have demonstrated that the daily poor gait performance was associated with the risk of falls (van Schooten et al., 2015), Parkinson’s disease (Weiss et al., 2014, 2015), and AD (Hsu et al., 2014). To the best of our knowledge, the association of daily gait abnormalities and cognition in patients with MCI, particularly the subtype of aMCI, has not been investigated using this technique.

In this study, we used an inertial-sensor-based wearable device to collect the daily gait data. We aimed to primarily explore the characteristic of gait disorder among the elderly with aMCI compared with the healthy elderly and secondarily determine the extent to which the daily gait deficit was quantitatively associated with cognition.

## Materials and Methods

### Study Setting and Participants

In all, 38 participants diagnosed with aMCI at neurological clinics at First People’s Hospital of Foshan between December 2017 and March 2018 were recruited. Cognitively normal controls (NC) with matching demographic information (age, gender, and education level) were enrolled from the communities. Ethics approval was obtained from the First People’s Hospital of Foshan Research Ethics Board, and written informed consent was obtained from the participants at enrollment.

All the participants were enrolled according to the following inclusion criteria: aged 55 years or older and able to walk independently without any gait assistance. The aMCI diagnostic criteria (Petersen, 2004) were as follows: (1) subjective cognitive complaint, preferably confirmed by an informant; (2) a single domain or multi-domain cognitive decline, and abnormal objective memory impairment identified by a cutoff of 1.5 SD below age and education matched-specific norms on a memory test; (3) preserved activities of daily living confirmed by a clinician’s interviews; (4) absence of dementia according to Diagnostic and Statistical Manual of Mental Disorders, Fourth Edition, revised (DSM-IV-R); and (5) global Clinical Dementia Rating (CDR) = 0.5 (Morris, 1993). The inclusion criteria for NC participants included: (1) cognitively normal, with no memory complaints or memory difficulties, verified by an informant; and (2) CDR = 0.

The exclusion criteria for all the participants included any neurologic disorder and other systematic disease that would likely contribute to cognitive and/or motor deficits (history of stroke, Parkinson’s disease, epilepsy, brain trauma, hypertension, diabetes, small vessel disease confirmed by MRI, etc.), active rheumatic and orthopedic diseases that affect lower limbs, history of knee/hip replacement, etc.), use of neuroleptics or benzodiazepines, and psychiatric comorbidity (e.g., major depressive disorder determined by the DSM-IV-R criteria).

### Outcome Measures

#### Medical and Cognitive Assessment

Demographic characteristics, medical history, history of falls, and activities of daily living were collected using standardized questionnaires during face-to-face interviews. The study clinicians performed a physical examination including a neurological examination on all participants.

Global cognition was assessed using the Mini-Mental State Examination (MMSE; Folstein et al., 1975) and the Chinese version of Montreal Cognitive Assessment (MoCA; Chen et al., 2016). A neuropsychological test battery was administered. The executive function was assessed using Stroop’s Color Word Test (SCWT); memory was assessed using The Auditory Verbal Learning Test-Huashan version (AVLT-H; Guo and Hong, 2001); language was assessed using the Boston Naming Test; and attention was assessed using the Symbol Digit Modalities Test (SDMT).

#### Gait Assessments

Gait data were collected by a wearable devise which we designed for gait measurement. Detailed experimental design, algorithm for gait parameters and validated method were reported in our previous study (Tao et al., 2018). Briefly, it comprised wearable devices of shoes and modules with the inertial Micro-Electro-Mechanical System (MEMS) sensors fixed under the shoe heel bottom, collecting motion signals and transmitting them to a computer. The high-order low-pass filter and hexahedral calibration technique were employed in data preprocessing, which reduces high-frequency noise interference and installation errors produced by sensor devices. Moreover, accumulative errors were also corrected based on the zero-correction algorithm. The final gait parameters were obtained by fusing acceleration data and posture, which is calculated using quaternary complementary filtering technique.

Gait data were collected while walking and transmitted to a computer server. Sampling frequency was set to 20 Hz, which was sufficient to continually collect data while walking. Gait parameters collected included the total number of steps, walking velocity, stride length, stride time, cadence, and stride time variability [STV, the percentage of coefficient of variation (%CoV)]. The STV was calculated as follows:

$$STV(\text{CoV\%})=\frac{\sqrt{\frac{1}{m}\sum_{i=1}^{m}{\left(t_{i}-\bar{t}\right)}^{2}}}{\bar{t}}\cdot 100\%$$

In this formula, *m* denotes the number of total strides. *t*_i_ denotes the stride time of the ith stride. $\bar{t}$ denotes the average stride time of the total *m* strides.

All subjects were required to follow these procedures: (1) wear the inertial-sensor-based smart shoes for at least 1 h or more every day; (2) wear it for 2 weeks; (3) keep daily normal outdoor walking while wearing the smart shoes; (4) all subjects were forbidden to wear the smart shoes to carry out non-normal outdoor walking (such as sports, dancing, and indoor walking); and (5) under special circumstances (such as rain and snow weather and shoes failure detected by the terminal), the user is informed not to wear the smart shoes temporarily.

### Data Analysis

All the data collected in this study were statistically analyzed using SAS 9.2 (SAS Institute, Cary, NC, USA). The characteristics were compared between groups using Student’s *t*-test or Wilcoxon rank sum test for continuous variables, and Pearson’n *χ*^2^ test or Fisher’h exact test for the categorical variables. All the *p*-values < 0.05 of statistical tests were considered to be statistically significant.

## Results

Table 1 presents population characteristics of the sample in terms of the demographic variables and neuropsychological tests. No significant difference was observed in age, gender, and education level between two groups (*p* < 0.05). Scores of the MMSE, MoCA, and AVLT-H in aMCI group were significantly lower than those in normal group (*p* < 0.01). No significant difference was observed in Boston naming test and Stroop color and word test scores between two groups (*p* > 0.05).

**Table 1:** Group differences in demographic and clinical characteristics.

		(*n* = 30)	(*n* = 38)	*P*-value
Demographic variables	Age, year	68.83 ± 4.41	66.42 ± 5.04	0.17
	Male, *n* (%)	14 (46.7%)	16 (42.12%)	0.68
	Education, year	8.46 ± 1.19	8.64 ± 1.01	0.35
Hyperlipidemia	*n* (%)	7 (23.3%)	8 (21.1%)	0.56
Coronary artery disease	*n* (%)	2 (6.6%)	2 (5.2%)	0.32
Osteoporosis	*n* (%)	13 (43.3%)	16 (42.1%)	0.61
Obesity	*n* (%)	6 (20.0%)	8 (21.1%)	0.56
Smoking	*n* (%)	9 (30%)	11 (28.9%)	0.32
Mild depression	*n* (%)	2 (6%)	4 (10.1%)	0.08
Antidepressants	*n* (%)	2 (6%)	3 (7.8%)	0.23
**Cognitive tests**				
Memory	AVL-SR score	6.36 ± 2.46	3.87 ± 1.46	0.010*
	AVL-LR score	6.273 ± 2.17	4.33 ± 1.23	0.019*
Attention	Symbol Digit Modalities Test score	35.91 ± 7.24	32.55 ± 5.32	0.213
Language	Boston Naming score	21.64 ± 4.32	20.18 ± 5.02	0.113
Executive function	Stroop color word test score	2.15 ± 1.06	2.08 ± 0.77	0.849
	MMSE total score	26.87 ± 1.88	23.75 ± 2.52	<0.001**
	MoCA total score	24.13 ± 2.41	21.42 ± 2.02	<0.001**

*P < 0.05, **P < 0.01. AVL-SR: AVLT-H test, short-term (5-min delay time) delayed recalls. AVL-LR: AVLT-H test, long-term (20-min delay time) delayed recall.

As shown in Table 2, stride length (*p* < 0.05) and walking velocity (*p* < 0.01) in aMCI group were lower than those in the NC group. The stride time variability in the aMCI group was higher than that in the normal group (*p* < 0.05). The total number of steps, stride time, and cadence did not differ between two groups (*p* > 0.05).

**Table 2:** Characteristic of gait patterns between groups.

	NC (*n* = 30)	aMCI (*n* = 38)	*P*-value
Total step numbers	46,743 ± 29,248	33,747 ± 23,854	0.402
Walking velocity, m/s	1.14 ± 0.08	0.96 ± 0.12	0.008**
Stride length, m	1.19 ± 0.13	1.03 ± 0.20	0.033*
Stride time, seconds	1.12 ± 0.07	1.06 ± 0.08	0.054
Stride time variability (%)	3.18 ± 0.96	4.06 ± 0.98	0.026*
Cadence, steps/min	109.56 ± 6.37	107.98 ± 7.87	0.418

*P < 0.05, **P < 0.01.

A correlation analysis was conducted for all subjects to examine the correlations between cognitive domain and gait parameters. The results (Table 3) indicated that the walking velocity was positively associated with long-term delayed recall and executive function (*p* < 0.05). The stride length was positively associated with cognitive domains including long-term delay recall, executive function, and attention (*p* < 0.05). The stride time variability was negatively correlated with cognitive domains including long-term delay recall, executive function, and attention (*p* < 0.05).

**Table 3:** Correlations of cognitive domains between gait patterns for all subjects.

		Memory				
(*n* = 68)	AVL-SR	AVL-LR				
				Executive	Language	Attention
Mean ± SD	4.97 ± 2.36	5.19 ± 1.65	4.19 ± 2.11	2.11 ± 1.06	20.82 ± 4.74	32.36 ± 6.06
Total step numbers	−0.021	0.105	0.154	−0.129	0.121	−0.065
*P*-value	0.922	0.473	0.303	0.362	0.387	0.760
Walking velocity	0.021	0.256	0.125	0.49	0.112	0.145
*P*-value	0.877	0.043*	0.055	0.007**	0.280	0.109
Stride length	0.020	0.322	0.210	0.366	0.071	0.264
*P*-value	0.792	0.036*	0.169	0.024*	0.495	0.048*
Stride time variability	0.011	−0.206	−0.103	−0.430	−0.162	−0.247
*P*-value	0.964	0.038*	0.223	0.022*	0.385	0.015*
Cadence	−0.091	−0.086	−0.054	0.190	0.045	0.152
*P*-value	0.632	0.681	0.799	0.160	0.831	0.309

*P < 0.05, **P < 0.01. AVL-SR: AVLT-H test, short-term (5-min delay time) delayed recalls. AVL-LR: AVLT-H test, long-term (20-min delay time) delayed recall. Bold values indicated the correlation between memory and gait variables is significant.

## Discussion

Our findings show that among the aMCI group, stride length and the walking velocity were lower, while stride time variability was higher than those in the normal group, suggesting that aMCI adversely affected the gait performance. In addition, cognitive domains including memory, executive function, and attention for all the subjects correlated with the three gait parameters (stride length, walking velocity, and stride time variability), indicating that cognitive impairment was parallel to the gait abnormality.

Gait parameters such as velocity, stride time, and stride length are basic components reflecting rhythmic control and dynamic postural control while walking. Previous studies have demonstrated that these parameters were related to the occurrence of many diseases. Among them, the relationship between cognitive impairment and gait disorder has received increasing attention among individuals with MCI. Evidence has indicated that gait disorder with lower velocity and stride length may have occurred before the onset of dementia (Montero-Odasso et al., 2017; Sakurai and Montero-Odasso, 2017). Gait abnormality could be used as a surrogate marker for screening cognitive impairment in the early stage of AD and reflecting the progression of MCI into AD (Allali et al., 2016). In our study, sensor-based wearable devices were used to evaluate the gait performance. The results in this study were similar to those of previous studies on MCI. In addition, we recruited subjects with aMCI, an important subtype of MCI that is more closely related to the etiology of AD, which reduces the interference or bias of the heterogeneity of MCI on the experimental results. Our results provided further evidence that gait as a biomarker could be useful for indicating the early stage of AD.

Apart from velocity, stride time, and stride length, stride time variability is an important spatiotemporal parameter that measures gait stability from stride to stride. It reflects the ability to regulate and maintain stable gait patterns. It has been reported that increased stride time variability was associated with the prodromal stage of AD (Allali et al., 2016; Beauchet et al., 2019) and was recognized as a specific biomarker for the identification of MCI (Beauchet et al., 2018), particularly under a dual-task condition (Boripuntakul et al., 2014; Montero-Odasso et al., 2017). Our results showed that stride time variability in aMCI patients (4.06 ± 0.98%) was higher than that in normal subjects (3.18% ± 0.96%). Consistent with previous studies, the results of this study provided further evidence that increased stride time variability could be used to distinguish aMCI patients from normal healthy elderly. Importantly, this study used built-in sensors and wearable devices to measure mobility from daily life walking, instead of from a conventional one-time clinical assessment, which only allows for collecting data for a short period of time, thus providing evidence for an easy-to-use and objective measurement for the identification of individuals at the risk of dementia.

Our study also confirmed the results in previous studies in which the performance of the cognitive domains in terms of memory, executive function, and attention was closely related to gait parameters. The pathological mechanism of gait dysfunction in the MCI population has not been fully understood thus far. Previous studies have demonstrated that the relationship between cognitive impairment and gait abnormalities may be attributed to the disruption of the functional connectivity of a neural network that is jointly responsible for cognition and controlling walking (Rosano et al., 2012; Crockett et al., 2017) The underlining pathological mechanism is the AD-related degenerative lesions in the brain regions, particularly involved in the temporal and the prefrontal lobes. The lesions accumulate and gradually impair the areas related to the cognitive and motor functions, leading to both abnormal cognitive abilities and gait disorders (Del Campo et al., 2016).

## Limitations

This study had some potential limitations. The small sample size of this study (*n* = 68) might reduce the statistical efficacy of this study and affect the accuracy of the conclusions. In addition, this study did not recruit non-amnestic subjects. Thus, the results did not characterize the patterns for the different subtypes of MCI. Furthermore, given the lack of etiology evidence of AD from well-established biomarkers, we were unable to determine whether or not subjects with aMCI in this study were MCI due to AD. Evidence from previous studies demonstrated that individuals with aMCI were at a relative high risk of developing into AD. However, aMCI was a heterogeneous population. Some may develop into other type of dementia. Future studies should be conducted to clarify the value of sensor-based gait measurement applied to screening early AD and MCI due to AD.

## Conclusion

This study suggested that cognitive impairment-related gait disorders occur (reduced gait speed, gait length, and gait stability) in daily life walking among the aMCI patients. A sensor-based wearable device for gait measurement may be an alternative and convenient tool for screening cognitive impairment. To the best of our knowledge, this study is the first to provide supporting evidence that gait sensor-based wearable devices for gait measurements may be an alternative and easy-to-use tool for detecting the transition phases leading to AD and tailoring early intervention. Future studies should further explore the specificity and the accuracy of gait parameters to distinguish the aMCI patients from normal healthy elderly and predict future cognitive decline.

## Data Availability Statement

The datasets generated for this study are available on request to the corresponding author.

## Ethics Statement

The studies involving human participants were reviewed and approved by First People’s Hospital of Foshan Research Ethics Board. The patients/participants provided their written informed consent to participate in this study.

## Author Contributions

HX and ZL contributed to the conception of the study and design, study supervision, interpretation of data and manuscript preparation. YW and ST helped perform the analysis and critical revision of the manuscript. SH and CZ contributed to acquisition of data and data analyses.

## Conflict of Interest

The authors declare that the research was conducted in the absence of any commercial or financial relationships that could be construed as a potential conflict of interest.
